# A Nomogram for Predicting Tenofovir-Associated Osteoporosis in Chronic Hepatitis B

**DOI:** 10.3390/jcm15124442

**Published:** 2026-06-09

**Authors:** Elif Can Semet, Cihan Semet

**Affiliations:** 1 Department of Physical Medicine and Rehabilitation, Bandırma Training and Research Hospital, Balıkesir 10200, Türkiye; 2 Department of Infectious Diseases and Clinical Microbiology, Faculty of Medicine, Bandırma Onyedi Eylül University, Balıkesir 10250, Türkiye

**Keywords:** chronic hepatitis B, tenofovir disoproxil fumarate, osteoporosis, clinical prediction model, nomogram, LASSO regression, DXA screening, decision curve analysis

## Abstract

**Background/Objective:** Long-term tenofovir disoproxil fumarate (TDF) therapy is associated with progressive bone mineral density loss in patients with chronic hepatitis B (CHB), yet existing fracture risk algorithms, such as FRAX, were not designed for this population. We aimed to develop and internally validate a clinical nomogram for identifying TDF-associated osteoporosis using penalized regression on demographic, virological, and biochemical predictors. **Methods:** In this single-center retrospective cohort study, 237 adult CHB patients receiving TDF for at least 12 months underwent dual-energy X-ray absorptiometry (DXA). Osteoporosis was defined as a T-score of −2.5 or lower at the lumbar spine or femoral neck. Thirteen candidate predictors were evaluated using LASSO regression with 10-fold cross-validation; selected variables were entered into an unpenalized multivariable logistic regression model; internal validation employed bootstrap resampling with 200 replications to derive optimism-corrected estimates of discrimination and calibration. The clinical utility was assessed using decision curve analysis (DCA). **Results:** Osteoporosis prevalence was 15.2% (n = 36). LASSO selected three predictors: prior fragility fracture (OR 11.45, 95% CI 4.82–27.15), the Charlson Comorbidity Index (OR 1.45 per unit, 95% CI 1.15–1.85), and alkaline phosphatase. The model demonstrated strong discrimination (apparent C-index 0.860; optimism-corrected 0.845) with excellent calibration (slope 0.94, intercept 0.02; Brier score 0.095). At a 0.15 probability threshold, sensitivity was 86.0%, specificity 78.0%, and negative predictive value 97.0%. DCA confirmed superior net clinical benefit over default strategies across the 0.10–0.30 threshold range; a pre-specified sensitivity analysis excluding fracture history retained meaningful discrimination (corrected C-index 0.791). **Conclusions:** This nomogram offers a clinically actionable, disease-specific tool for stratifying osteoporosis risk in TDF-treated CHB patients, particularly well suited for safely deferring DXA imaging in low-risk individuals. External validation in multicenter and ethnically diverse cohorts is required before widespread implementation.

## 1. Introduction

Chronic hepatitis B (CHB) remains a major global health burden, requiring long-term viral suppression with nucleos(t)ide analogs to prevent disease progression to cirrhosis and hepatocellular carcinoma [1]. Tenofovir disoproxil fumarate (TDF) has long served as a cornerstone of CHB therapy owing to its high genetic barrier to resistance and potent antiviral efficacy [2]. However, prolonged TDF administration is increasingly associated with off-target metabolic complications, most notably a progressive decline in bone mineral density (BMD) that may culminate in osteoporosis and fragility fractures [3]. This phenomenon unfolds within the broader context of hepatic osteodystrophy, creating a complex clinical dilemma of balancing durable virological control against the risk of iatrogenic skeletal morbidity. As the CHB population ages and remains on therapy for decades, early identification of patients at highest risk for TDF-induced bone loss becomes a clinical priority to enable timely therapeutic optimization or transition to alternative antiviral regimens.

Current understanding of TDF-associated osteotoxicity suggests a multifactorial etiology involving proximal renal tubular dysfunction, altered phosphate homeostasis, and direct interference with osteoblast activity [4]. Analogous mechanisms have been described in TDF-treated populations beyond CHB; recent work in young adults living with HIV has identified prolonged TDF exposure, alongside age, body mass index, and viral markers, as a leading contributor to bone mineral density decline, and machine-learning approaches have been proposed to stratify individual risk in resource-limited settings in which DXA access is restricted [5]. Comparable findings in male patients with HIV/AIDS receiving TDF-containing antiretroviral therapy have further linked treatment duration and comorbid factors to bone mass reduction, while also highlighting the contribution of psychological burden and the value of formal risk prediction in supporting early intervention [6]. While clinicians frequently rely on traditional risk factors (such as age, body mass index, and menopausal status) to screen for osteoporosis, these parameters often fail to capture the unique pathophysiological contributions of chronic viral infection and systemic inflammation [7]. Evidence remains conflicting regarding the precise roles of viral load suppression, duration of TDF exposure, and liver fibrosis severity in accelerating BMD loss [8]. Moreover, conventional fracture risk assessment tools were primarily developed for the general population and may underperform in the specific context of CHB patients receiving long-term TDF [9]. Consequently, real-world data are scarce regarding how to integrate hepatitis B virus (HBV)-specific virological parameters with markers of systemic inflammation to predict individual skeletal outcomes accurately.

A critical research gap persists in the management of TDF-associated bone disease in CHB, with three specific deficiencies in the current evidence base remaining unresolved. First, no validated, disease-specific risk-stratification tool currently exists for identifying CHB patients at greatest risk of TDF-associated osteoporosis; existing fracture-risk algorithms such as FRAX were derived from general-population cohorts and predict incident fracture rather than prevalent low BMD, leading to a conceptual mismatch with the screening question encountered in routine hepatology practice. Second, although systemic inflammation, hepatic fibrosis, and cumulative comorbidity are biologically plausible contributors to bone loss in CHB, no published prediction model has formally integrated such markers (e.g., the SII, FIB-4, APRI, and CCI) alongside conventional demographic and biochemical predictors within a single, internally validated framework. Third, traditional unpenalized multivariable models often struggle with collinearity and overfitting when many candidate variables are evaluated in cohorts with a limited number of events, particularly for an outcome as low-prevalence as DXA-defined osteoporosis. The present study addresses all three gaps directly: it develops a CHB-specific predictive framework, incorporates inflammatory and fibrosis biomarkers (SII, FIB-4, APRI) alongside the CCI within the candidate predictor pool, and applies penalized variable selection via the Least Absolute Shrinkage and Selection Operator (LASSO) regression to identify the most robust predictors of bone loss from a comprehensive dataset while explicitly mitigating overfitting. The study was conducted at a tertiary-care university hospital with standardized long-term antiviral follow-up between 2020 and 2025, a period that reflects contemporary CHB management and provides a real-world cohort with sufficient cumulative TDF exposure to evaluate therapy-related skeletal complications.

The primary objective of this study was to develop and internally validate a predictive nomogram for osteoporosis among adult patients with CHB receiving TDF-based therapy. We hypothesized that a model derived from a comprehensive set of demographic, virological, biochemical, and inflammatory candidate predictors, using a penalized regression framework, would demonstrate superior discriminative ability and clinical utility for identifying TDF-associated osteoporosis compared with standard clinical assessments [10]. The primary outcome was osteoporosis, defined as a T-score of −2.5 standard deviations or lower at the lumbar spine or femoral neck on dual-energy X-ray absorptiometry (DXA), in accordance with World Health Organization (WHO) criteria [11]. Secondary objectives included evaluating calibration and net clinical benefit via Decision Curve Analysis (DCA). To this end, we conducted a retrospective cohort study at a tertiary-care university hospital between 2020 and 2025, with data partitioned into training and validation sets to assess the robustness and generalizability of the predictive model.

## 2. Materials and Methods

### 2.1. Study Design and Patient Population

This retrospective, observational cohort study was conducted at a tertiary-care university hospital between 2020 and 2025. The study protocol was approved by the Institutional Review Board of Bandırma Onyedi Eylül University Non-Interventional Clinical Research Ethics Committee (Decision No. 2026-04-07, 28 April 2026), and the requirement for informed consent was waived due to the study’s retrospective design. The study was conducted in accordance with the principles of the Declaration of Helsinki. This study is reported in accordance with the Transparent Reporting of a multivariable prediction model for Individual Prognosis or Diagnosis (TRIPOD) statement [10]. In line with TRIPOD recommendations for a model development study with internal validation (TRIPOD type 1b), we pre-specified the data source, candidate predictor set, outcome definition, sample size considerations, and procedures for handling missing data, variable selection, internal validation, and assessment of model performance across the three complementary dimensions of discrimination, calibration, and clinical utility. The model intercept and regression coefficients of all retained predictors are reported in full to permit external use and validation, and the final model is presented as a nomogram to facilitate bedside application. The completed TRIPOD checklist, with item-by-item mapping to the corresponding sections of the present manuscript, is provided as Supplementary Materials Table S2.

We screened adult patients (aged 18 years or older) diagnosed with chronic HBV infection who had been receiving TDF-based nucleos(t)ide analogue therapy for a minimum of 12 continuous months. To be included, patients were required to have at least one valid DXA scan performed during routine follow-up. Patients were excluded if they had human immunodeficiency virus (HIV) co-infection, pre-existing metabolic bone diseases (including Paget’s disease or osteomalacia), chronic kidney disease (stage 4 or 5) prior to antiviral initiation, malignancies with bone metastasis, or a history of anti-osteoporotic treatment (including bisphosphonates or denosumab) prior to baseline DXA evaluation, in order to minimize confounding effects on BMD.

### 2.2. Data Collection and Variable Definition

Clinical and demographic data were systematically extracted from the electronic hospital information system. Variables included age, sex, body mass index (BMI), smoking status, alcohol consumption, menopausal status, and the Charlson Comorbidity Index (CCI), which was calculated for each patient using the standardized scoring algorithm to quantify comorbidity burden [12]. HBV-specific virological parameters included the duration of TDF exposure (months), viral load suppression status (HBV DNA), hepatitis B e-antigen (HBeAg) status, and hepatitis C virus (HCV) co-infection. To reflect the severity of underlying hepatic impairment, two non-invasive liver fibrosis markers were calculated using routine laboratory values: the FIB-4 index, computed as (Age × aspartate aminotransferase [AST])/(Platelet count × sqrt alanine aminotransferase [ALT]) [13], and the APRI, computed as (AST/Upper Limit of Normal)/Platelet count × 100 [14]. Additional laboratory parameters recorded closest to the date of the DXA scan included serum calcium, phosphorus, alkaline phosphatase (ALP), 25-hydroxyvitamin D, intact parathyroid hormone (PTH), and estimated glomerular filtration rate (eGFR). To assess systemic inflammatory burden, the SII was calculated as (Neutrophil count × Platelet count)/Lymphocyte count [15]. Exposure to osteotoxic medications, including systemic corticosteroids and proton pump inhibitors (PPIs), was also recorded.

### 2.3. Outcome Measurement

The primary outcome was the presence of TDF-associated osteoporosis as established by DXA. All scans were performed using a single, manufacturer-calibrated DXA system (Primus; OsteoSys Co., Ltd., Seoul, South Korea) at our institution, with daily quality-control phantom scans to monitor instrument stability and prevent measurement drift across the study period. BMD was measured at two anatomically standardized regions of interest: the anteroposterior lumbar spine (L1–L4) and the femoral neck of the non-dominant hip. Vertebrae demonstrating severe focal degenerative changes, prior surgical instrumentation, or compression deformities that would invalidate BMD measurement at the affected level were excluded from analysis at that specific site, with the remaining valid measurements used for diagnostic classification. T-scores were calculated by comparing each patient’s BMD to the manufacturer-provided young-adult reference population matched for sex, expressed as the number of standard deviations from the mean peak BMD of a healthy young-adult reference. In accordance with World Health Organization criteria, osteoporosis was defined as a T-score of −2.5 standard deviations or lower at either the lumbar spine or femoral neck [11]; the lowest valid T-score across the two regions was used to classify each patient as having or not having osteoporosis. All DXA scans were interpreted by an experienced clinician blinded to antiviral exposure history, treatment duration, and the laboratory parameters used as candidate predictors in the present model.

### 2.4. Penalized Regression-Based Variable Selection and Statistical Analysis

Sample size considerations followed current recommendations for developing clinical prediction models [16]. The available cohort of 237 patients with 36 osteoporosis events yielded an events-per-variable (EPV) ratio of 2.8 for the 13 initial candidate variables, which would have been insufficient for unpenalized multivariable regression. To address this constraint, LASSO regression was selected as a penalized variable selection method explicitly designed to mitigate overfitting in low-EPV settings. The final model included three predictors, yielding an effective EPV ratio of 12.0, which exceeds the minimum threshold commonly recommended for stable parameter estimation in unpenalized logistic regression.

The analytic dataset was constructed using a complete case approach. Patients with missing values for any of the candidate demographic, virological, or laboratory variables (including 25-hydroxyvitamin D, alkaline phosphatase, eGFR, and the hematological and biochemical components required to derive the Systemic Immune-Inflammation Index, FIB-4, and APRI) were excluded during the initial screening phase. The final cohort of 237 patients had no missing data for any candidate variable. No imputation procedures were therefore required.

Prior to penalized regression, multicollinearity among the 13 candidate variables was assessed using generalized variance inflation factors (VIF) derived from a full multivariable logistic regression model. All VIF values were below the conventional threshold of concern (highest VIF = 5.42 for age; mean VIF = 2.56), indicating that the candidate predictor set did not exhibit substantial collinearity. The complete VIF table is provided as Supplementary Materials Table S1. Variables with overlapping clinical content (e.g., age, comorbidity burden, and TDF exposure duration) showed moderate but acceptable interrelationships, supporting the appropriateness of LASSO regression for parsimonious feature selection without prior variable elimination on collinearity grounds.

The LASSO regression method was applied to the entire cohort to perform penalized variable selection and minimize overfitting when evaluating multiple candidate clinical variables [17]. LASSO was deliberately preferred over alternative dimensionality-reduction approaches such as principal component analysis (PCA) because the analytical objective was to derive a clinically interpretable nomogram for bedside use rather than to reduce the candidate variable set to abstract orthogonal components. PCA generates linear combinations of original predictors that maximize explained variance but lose direct clinical interpretability and complicate the construction of a transparent risk-scoring tool; in contrast, LASSO retains the original measurement scales of the selected variables and produces coefficients that can be reported and applied in routine clinical practice. The optimal penalization coefficient (lambda) was determined using 10-fold cross-validation, and the one-standard-error rule (1-SE rule) was applied to select the most parsimonious model [18]. Variables with non-zero coefficients in the LASSO model were subsequently entered into an unpenalized multivariable logistic regression model to construct the predictive nomogram and obtain interpretable odds ratios.

Internal validation was performed using bootstrap resampling with 200 replications, in line with current methodological recommendations for clinical prediction models [19,20]. This approach uses all available observations for both model development and validation and yields optimism-corrected estimates of model performance, offering greater statistical efficiency than split-sample validation for cohorts of this size. Model performance was evaluated across three complementary dimensions: discrimination (apparent and optimism-corrected concordance index, mathematically equivalent to the area under the receiver operating characteristic curve [AUC] for binary outcomes), calibration (calibration slope, calibration intercept, and Brier score, visualized with bootstrap-corrected calibration plots), and clinical utility. Clinical utility was evaluated using DCA, which estimates the net benefit of the nomogram across a range of threshold probabilities and allows comparison with the default strategies of treating all or no patients [21]. The probability threshold for binary classification (0.15) was selected using a hybrid approach that combined a statistical optimum derived from the Youden index with clinical considerations, with preference for maximizing negative predictive value to support the safe deferral of DXA imaging in low-risk patients. To address potential concerns about reverse causality, given that prior fragility fractures may themselves reflect prevalent rather than incident osteoporosis, a prespecified sensitivity analysis was performed by refitting the model after excluding fracture history, with discrimination compared between the full and reduced models using DeLong’s test [22]. All statistical analyses were performed using R software (version 4.3.0; R Foundation for Statistical Computing, Vienna, Austria) with the rms (version 6.7-0), glmnet (version 4.1-7), and pROC (version 1.18.5) packages.

## 3. Results

### 3.1. Cohort Characteristics and Osteoporosis Prevalence

**Figure 1:**
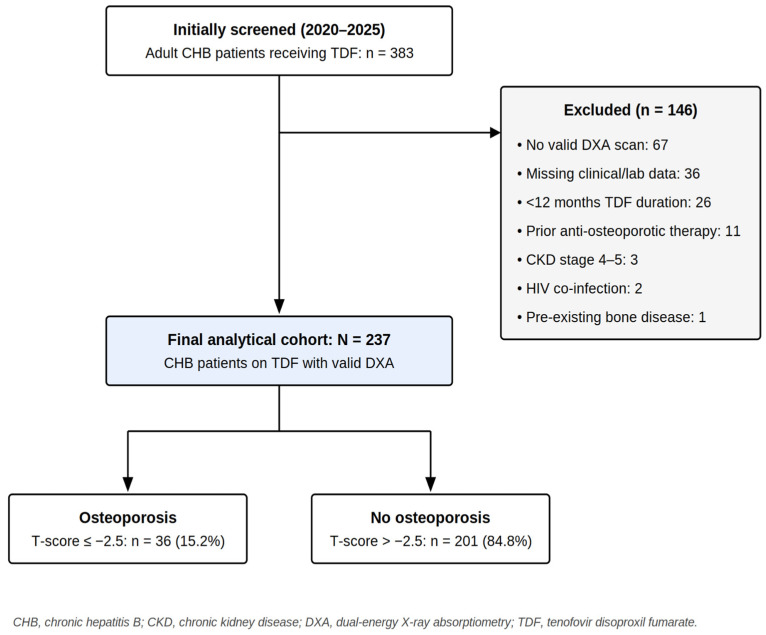
Patient flow diagram.

A total of 237 adult patients with chronic hepatitis B receiving TDF-based therapy were included in this study. The mean age of the cohort was 50.8 ± 11.9 years, and 48.5% were male. Osteoporosis (T-score of −2.5 or lower) was diagnosed in 36 patients (15.2%) based on DXA scan results, with a numerical preponderance among male patients (21/115, 18.3%) relative to female patients (15/122, 12.3%); this between-sex difference did not reach statistical significance (*p* = 0.272). Postmenopausal status was disproportionately concentrated within the osteoporosis subgroup (38.9% vs. 21.9%, *p* = 0.048), and nearly all female osteoporosis cases (14/15) were classified as postmenopausal, consistent with the well-established interaction between advanced age, sex hormone deficiency, and skeletal fragility in this population. The patient selection process is summarized in Figure 1.

Table 1 presents the baseline characteristics of the study population stratified by osteoporosis status. Patients with osteoporosis were significantly older (64.6 ± 9.7 vs. 48.3 ± 10.4 years, *p* < 0.001), had lower BMI (23.3 ± 2.1 vs. 26.3 ± 2.2 kg/m^2^, *p* < 0.001), and higher CCI scores (2.5 ± 1.2 vs. 0.7 ± 1.0, *p* < 0.001). A history of fragility fracture was significantly more prevalent in the osteoporosis group (50.0% vs. 7.0%, *p* < 0.001), corresponding to a more than seven-fold relative difference in fracture prevalence between groups.

Regarding HBV-specific parameters, patients with osteoporosis had significantly longer TDF exposure duration (85.0 ± 27.9 vs. 40.8 ± 26.5 months, *p* < 0.001) and higher rates of HCV co-infection (25.0% vs. 6.5%, *p* = 0.001). Laboratory parameters showed significant differences, with the osteoporosis group demonstrating elevated ALP (179.7 ± 49.3 vs. 106.6 ± 40.8 U/L, *p* < 0.001), higher SII (600.7 ± 118.7 vs. 482.8 ± 75.9, *p* < 0.001), lower vitamin D levels (14.5 ± 6.8 vs. 25.9 ± 9.1 ng/mL, *p* < 0.001), and reduced eGFR (67.2 ± 16.9 vs. 94.6 ± 18.3 mL/min/1.73 m^2^, *p* < 0.001). PPI use was significantly more prevalent in patients with osteoporosis (83.3% vs. 26.9%, *p* < 0.001).

Flow chart of patient screening and inclusion. Of 383 adult chronic hepatitis B patients receiving tenofovir disoproxil fumarate therapy between 2020 and 2025, 146 were excluded for inclusion or exclusion criteria as detailed, yielding a final analytical cohort of 237 patients. Osteoporosis prevalence in the analytical cohort was 15.2%.

### 3.2. Predictors of TDF-Associated Osteoporosis Identified by LASSO

**Figure 2:**
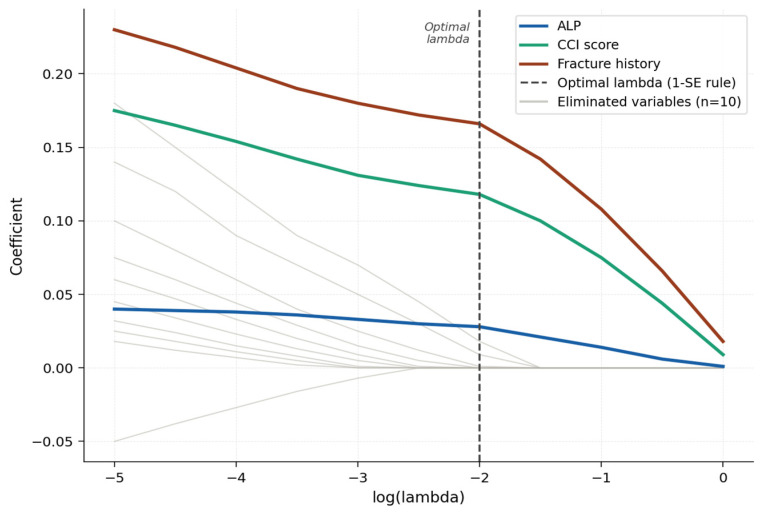
LASSO regularization coefficient path.

**Figure 3:**
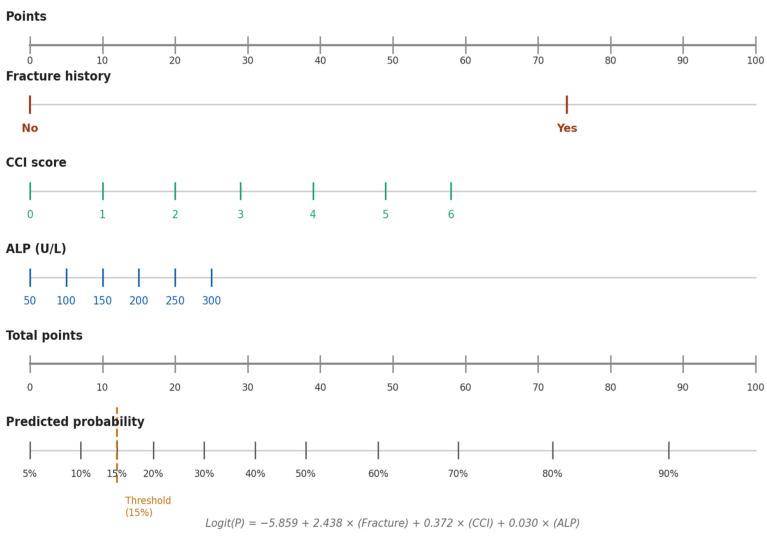
Clinical nomogram for individualized prediction of TDF-associated osteoporosis.

Using LASSO regression with 10-fold cross-validation and the one-standard-error rule, applied to the full cohort (N = 237), three predictors were selected from the initial 13 candidate variables (Figure 2) for inclusion in the final predictive model: CCI score, fracture history, and ALP level. To construct the predictive nomogram and obtain clinically interpretable odds ratios free of the shrinkage penalty inherent to the LASSO algorithm, these selected features were subsequently fit to an unpenalized multivariable logistic regression model. In this final framework, a history of fracture emerged as the strongest independent predictor of osteoporosis (OR = 11.45, 95% CI: 4.82–27.15). This magnitude strongly aligns with the substantial prevalence disparity observed in the baseline cohort (50.0% vs. 7.0%). Additionally, each unit increase in CCI score was associated with a 45% increase in the odds of osteoporosis (OR = 1.45, 95% CI: 1.15–1.85). Notably, while the unpenalized 95% confidence interval for ALP crossed 1.0 (OR = 1.03, 95% CI: 0.98–1.08) the variable was retained in the final nomogram. This retention is consistent with the foundational principle of LASSO selection, which optimizes global predictive performance through cross-validation rather than relying on isolated *p*-value thresholds [23], and it preserves a clinically meaningful biomarker of the liver–bone axis [24]. ALP captures unique pathophysiological information related to osteoblastic activity [25] and hepatic osteodystrophy [26] that is not redundantly conveyed by CCI or fracture history, supporting its inclusion despite a marginal standalone *p*-value. Table 2 presents the unpenalized coefficients and corresponding odds ratios with 95% confidence intervals for all three selected predictors. The resulting clinical nomogram is presented in Figure 3.

Coefficient trajectories for 13 candidate variables as a function of log(lambda). The dashed vertical line marks the optimal lambda (1-SE rule) at which three variables retain non-zero coefficients: ALP, CCI score, and fracture history.

The nomogram permits individualized risk estimation by assigning points to each predictor along its corresponding scale; the summed total points are then mapped to the lower probability axis to obtain the predicted probability of osteoporosis. The underlying model is: logit(P) = −5.859 + 2.438 × (fracture history) + 0.372 × (CCI score) + 0.030 × (ALP, U/L). The 0.15 probability cut-point indicated on the figure was selected a priori using a hybrid statistical–clinical strategy: the Youden index from the apparent ROC analysis identified a near-optimal cut-point in this region, while the clinical priority of safely deferring DXA imaging in low-risk patients required a threshold that maximized negative predictive value. At 0.15, the model achieved an NPV of 97.0%, meaning that approximately 97 of every 100 patients classified as low-risk by the nomogram were free of DXA-defined osteoporosis, supporting the use of this cut-point as a conservative referral threshold for routine clinical practice. Patients with predicted probabilities at or above 0.15 should be referred for DXA scanning; below this threshold, DXA can reasonably be deferred and rescheduled at the discretion of the treating clinician. Importantly, the 0.15 cut-point is not a fixed mandate: clinicians may select alternative thresholds along the decision-curve range (0.10–0.30) if local resources, patient preferences, or institutional protocols favor a more or less conservative screening strategy.

### 3.3. Discrimination, Calibration, and Classification Performance

**Figure 4:**
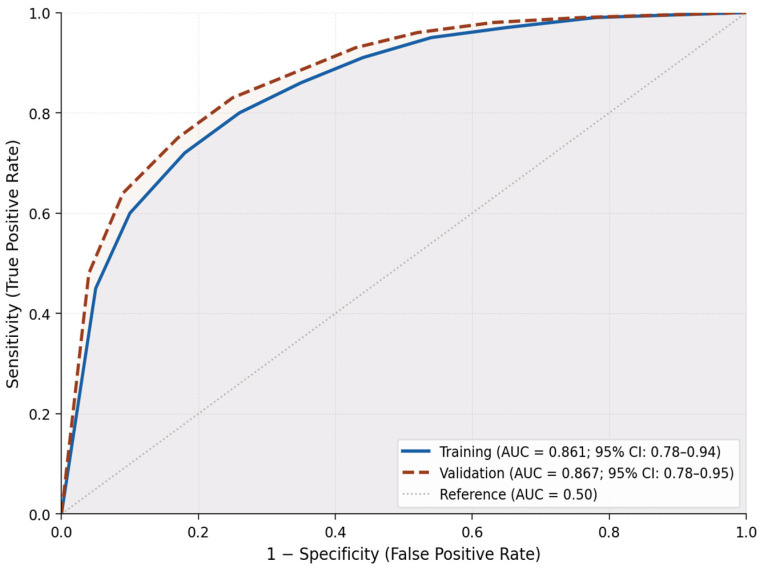
Receiver operating characteristic (ROC) curves.

**Figure 5:**
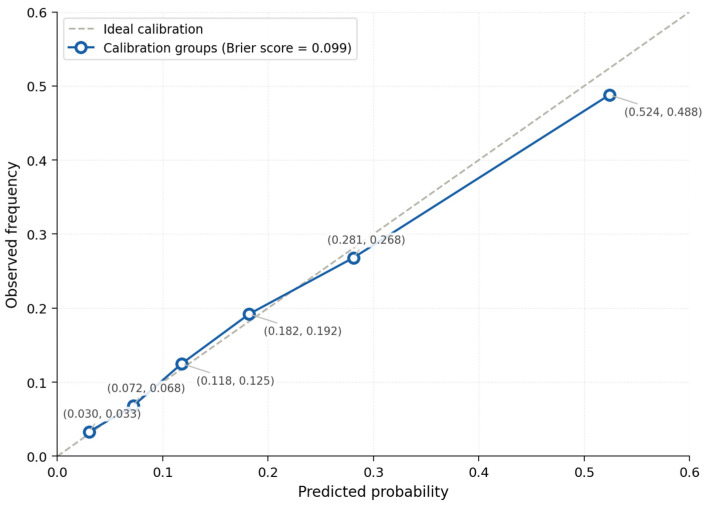
Calibration plot.

The predictive nomogram was developed using the full cohort (N = 237) and internally validated using bootstrap resampling with 200 replications. Table 3 summarizes the apparent and optimism-corrected performance metrics. The model demonstrated strong discriminative ability with an apparent C-index of 0.860 and an optimism-corrected C-index of 0.845, corresponding to a minimal optimism estimate of 0.015 and confirming the absence of substantial overfitting (Figure 4). Calibration was excellent: the bootstrap-corrected calibration slope was 0.94 (ideal value, 1.00) and the calibration intercept was 0.02 (ideal value, 0.00), with a Brier score of 0.095, indicating close agreement between predicted probabilities and observed outcomes (Figure 5). At a probability threshold of 0.15, the model achieved sensitivity of 86.0%, specificity of 78.0%, positive predictive value of 42.0%, and negative predictive value of 97.0%. The high negative predictive value indicates that the model is particularly well-suited for safely deferring DXA imaging in low-risk patients. The confusion matrix at the 0.15 threshold is presented in Table 4.

Receiver operating characteristic (ROC) curve Apparent ROC curve for the final logistic regression model in the full cohort (N = 237). Apparent C-index = 0.860; optimism-corrected C-index (bootstrap, B = 200) = 0.845. Diagonal represents chance-level discrimination.

Bootstrap-corrected calibration plot Bootstrap-corrected agreement between predicted probabilities and observed osteoporosis frequencies (B = 200 replications). Calibration slope = 0.94, calibration intercept = 0.02, Brier score = 0.095. Points near the ideal diagonal indicate good calibration.

### 3.4. Net Clinical Benefit of the Nomogram

**Figure 6:**
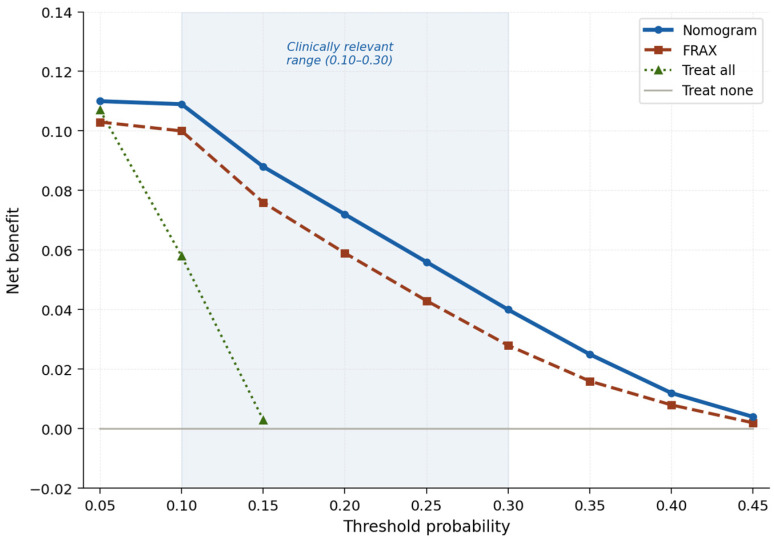
Decision curve analysis.

Decision Curve Analysis demonstrated that the nomogram provided superior net clinical benefit compared to both the treat-all and treat-none default strategies across a wide range of threshold probabilities (0.05–0.45). Within the clinically relevant range for DXA screening decisions (0.10–0.30), the nomogram consistently yielded greater net benefit than either default strategy, indicating that risk-stratified screening guided by the model would identify more true osteoporosis cases per unnecessary DXA performed than universal or no screening (Figure 6).

Decision curve analysis: Net clinical benefit of the nomogram across threshold probabilities, compared with the default strategies of treating all and no patients. The shaded band (0.10–0.30) marks the clinically relevant range for DXA screening decisions, within which the nomogram consistently outperforms both default strategies.

### 3.5. Sensitivity Analysis Excluding Fracture History

To address potential concerns about reverse causality, given that fragility fractures may themselves reflect prevalent osteoporosis rather than serve as independent predictors, a sensitivity analysis was performed by refitting the model with only the CCI and ALP as predictors. The reduced model retained clinically meaningful discriminative ability, with an apparent C-index of 0.805 (95% CI: 0.732–0.878) and an optimism-corrected C-index of 0.791. Although the difference in apparent C-index between the full model (0.861) and the reduced model (0.805) reached statistical significance (DeLong test, *p* = 0.031), the modest magnitude of the reduction (ΔC-index = 0.056) indicates that fracture history contributes meaningfully but is not the sole determinant of model performance. At the 0.15 probability threshold, the reduced model achieved a sensitivity of 75.0%, a specificity of 72.6%, a positive predictive value of 33.0%, and, notably, preserved a high negative predictive value of 94.1%, supporting its clinical utility for ruling out osteoporosis even in fracture-naïve patients.

## 4. Discussion

In this retrospective cohort study, we developed and internally validated a clinical nomogram designed to predict osteoporosis in patients with CHB receiving long-term TDF therapy. Applying penalized variable selection via LASSO regression, our model identified the CCI, fracture history, and ALP as the most robust predictors. The nomogram demonstrated strong discriminative ability with an AUC of 0.867 in the validation set. Although the Fracture Risk Assessment Tool (FRAX) achieved a numerically higher AUC in our cohort, DCA revealed that the nomogram provided superior net clinical benefit across clinically relevant threshold probabilities, underscoring the value of a disease-specific predictive tool over general population-based algorithms in the management of the metabolic bone disease associated with chronic viral infection and antiviral toxicity. These findings suggest that this nomogram can serve as a more clinically actionable instrument for optimizing DXA screening and guiding therapeutic decisions in this population.

The need for disease-specific risk-stratification tools in populations with chronic viral hepatitis has been increasingly recognized. Generic fracture-risk algorithms, such as FRAX, were developed and calibrated using general-population cohorts and designed to estimate the 10-year probability of incident osteoporotic fracture rather than the prevalence of established low bone mineral density [9,18]. This conceptual mismatch with the clinical question addressed here, namely the identification of patients with prevalent DXA-defined osteoporosis, limits the direct applicability of such tools to our setting. Beyond this design difference, generic algorithms inherently fail to capture the multifactorial pathophysiology of chronic viral hepatitis, which encompasses systemic inflammation, hepatic dysfunction, and direct drug-induced osteotoxicity [27,28]. Prior studies have demonstrated that conventional fracture-risk tools underestimate skeletal events in patients with chronic conditions such as cirrhosis and chronic kidney disease, misclassifying a clinically significant proportion of high-risk individuals [29,30]. Analogous limitations have been reported in HIV-positive cohorts on antiretroviral therapy, where standard algorithms consistently predicted fracture rates substantially lower than observed outcomes [31]. The present nomogram addresses these gaps by integrating comorbidity burden and a liver-bone axis biomarker (alkaline phosphatase) into a tool specifically calibrated for CHB patients on long-term TDF therapy [32,33], in line with the principle of population-specific model development [25].

The predictors selected by LASSO regression warrant specific discussion in the context of HBV-related bone metabolism. The strong independent association between CCI and osteoporosis reinforces the concept that cumulative health deficits, rather than viral parameters alone, drive skeletal fragility in aging CHB populations. This finding aligns with cohort data indicating that long-term TDF use is independently associated with higher fracture risk compared to entecavir, particularly among elderly patients with greater comorbidity burden [34]. Although recent literature has highlighted a direct correlation between circulating HBV DNA levels and BMD decline, suggesting that viral replication itself accelerates bone loss [35], our model selected ALP rather than HBV DNA as the third predictor. Elevated ALP likely reflects increased osteoblastic activity and disturbances of the liver-bone axis, both of which may be exacerbated by the systemic inflammatory milieu of chronic infection [36]. The retention of ALP by the LASSO algorithm, despite its marginal univariate significance, highlights its complementary role in capturing variance unexplained by comorbidity burden or fracture history. The LASSO 1-SE rule explicitly prioritizes parsimony alongside cross-validated predictive performance, which can preserve variables with modest individual effect sizes when they contribute incrementally to global model accuracy. The exclusion of HBV DNA from the final model is likely attributable to the high rate of viral suppression in our cohort (97.9%), which precluded adequate variability for detection of this association, as well as potential collinearity with age and comorbidity indices. Future model iterations in populations with lower suppression rates should explicitly include it [35]. Furthermore, newer antiviral agents such as tenofovir alafenamide (TAF) demonstrate substantially improved renal and bone safety profiles compared to TDF [37,38], and the anticipated transition of CHB populations toward TAF-based regimens may necessitate recalibration of predictive models in future cohorts.

Several baseline characteristics that differed markedly between groups warrant brief additional comment, even though they were not retained as independent predictors by LASSO. Vitamin D deficiency (mean 25-hydroxyvitamin D 14.5 vs. 25.9 ng/mL) and reduced eGFR (mean 67.2 vs. 94.6 mL/min/1.73 m^2^) were both substantially more pronounced in the osteoporosis subgroup, consistent with their established biological roles in skeletal homeostasis and with the well-recognized renal toxicity of long-term TDF therapy. Their absence from the final model reflects a methodological rather than a biological consideration: in the presence of strong predictors such as the CCI (which itself partially encodes renal and metabolic comorbidity) and advanced age (closely correlated with cumulative vitamin D insufficiency in this Mediterranean cohort), the additional independent variance contributed by these laboratory parameters did not survive the cross-validated 1-SE penalty. From a clinical standpoint, vitamin D status and renal function nevertheless remain modifiable or directly actionable elements of management in TDF-treated CHB patients and should be assessed and addressed routinely, independent of the nomogram’s classification output. Likewise, the marked disparity in prior fragility-fracture prevalence between groups (50.0% vs. 7.0%) confirms that, in routine practice, any prior low-trauma fracture in a TDF-treated patient should be treated as an independent indication for prompt DXA evaluation, regardless of the nomogram’s predicted probability.

From a methodological standpoint, the rigor of the study design supports the validity of these conclusions. LASSO regression was employed to mitigate overfitting and identify the most parsimonious set of predictors from a broad candidate pool, a technique increasingly recommended for prediction model development in clinical research [16,32]. Internal validation was performed using bootstrap resampling with 200 replications, yielding optimism-corrected estimates of model performance using all available observations, and is preferred over single-split-sample validation for cohorts of this size [19,20]. The minimal optimism observed (a 0.015 difference between the apparent and corrected C-indexes) confirms the absence of substantial overfitting. Model performance was evaluated across three complementary dimensions consistent with established reporting standards [10,33]: discrimination (apparent and optimism-corrected C-index), calibration (calibration slope, calibration intercept, and Brier score), and clinical utility (DCA). The bootstrap-corrected calibration slope of 0.94 and calibration intercept of 0.02 are close to ideal values, confirming the model’s reliability for absolute risk estimation across the full range of predicted probabilities. The DCA results are particularly informative, as they demonstrate that applying the nomogram to guide DXA screening yields a higher net benefit than either a universal screening or no-screening strategy across threshold probabilities of 0.10 to 0.30. This directly translates statistical performance into clinical value by quantifying the trade-off between avoiding unnecessary procedures and preventing missed diagnoses [21]. Although metrics such as Net Reclassification Improvement can provide complementary insights into model performance [39], DCA was prioritized as it directly addresses the clinical decision context of DXA referral.

The interpretation of fracture history as a predictor warrants careful consideration. Fragility fractures are themselves a clinical manifestation of advanced osteoporosis, and their inclusion in a predictive model raises legitimate concerns about reverse causality: the strong association observed (OR = 11.45) may partly reflect prevalent rather than incident disease. To address this concern, we performed a pre-specified sensitivity analysis after excluding fracture history from the model. The reduced model based on CCI and ALP retained clinically meaningful discrimination (optimism-corrected C-index 0.791) and preserved a high negative predictive value (94.1% at the 0.15 threshold), confirming that the nomogram remains useful even in patients without prior fragility fractures. This finding has direct clinical implications: patients with prior fragility fractures already have an established indication for DXA screening and pharmacological intervention, whereas the principal target population for risk stratification consists of fracture-naïve patients in whom early identification of skeletal vulnerability is most actionable. The full model is therefore best positioned as a comprehensive risk-stratification tool. In contrast, the reduced model may serve as an alternative in fracture-naïve cohorts where the clinical question is whether to initiate de novo DXA screening.

The primary clinical utility of this nomogram lies in optimizing resource allocation and supporting individualized patient management. DXA scanning remains the gold standard for diagnosing osteoporosis, but it is costly and not universally accessible in resource-limited settings [40]. Universal screening of all TDF-treated patients is neither practical nor cost-effective. By stratifying patients by predicted risk, the nomogram enables clinicians to safely defer imaging in low-risk individuals while concentrating diagnostic and therapeutic resources on those at intermediate or high predicted probability. For patients identified as high risk, timely initiation of calcium and vitamin D supplementation, lifestyle counseling, or bisphosphonate therapy should be considered [41]. In addition, a high nomogram score may provide objective support for transitioning from TDF to a bone-sparing regimen such as TAF or entecavir, in line with the growing consensus that treatment selection in CHB should weigh virological efficacy against extrahepatic safety [30].

Several limitations must be acknowledged. First, the retrospective design inherently carries a risk of selection bias, although rigorous exclusion criteria were applied to minimize confounding from pre-existing bone conditions. Second, the single-center nature of this study may limit generalizability to community-based or ethnically diverse settings; external validation in multicenter, geographically distinct cohorts is essential before widespread clinical implementation [42,43]. Third, while SII, FIB-4, and APRI were included as candidate predictors, they were not retained in the final model. Their limited independent contribution likely reflects shared variance with retained predictors, particularly the established association between systemic inflammation, hepatic dysfunction, and comorbidity burden captured by the CCI, rather than a true absence of biological relevance, as evidenced by the moderate VIF values observed for these variables in pre-selection analysis (Supplementary Table S1). Fourth, this study did not assess longitudinal BMD changes, and the predictive performance of the nomogram for incident fracture over time remains to be determined in prospective designs. Fifth, although sex, postmenopausal status, vitamin D, and eGFR were all included among the 13 candidate predictors, none was retained by the LASSO algorithm at the 1-SE optimum; this likely reflects their substantial shared variance with age and the CCI rather than a lack of biological importance, and a complete subgroup analysis stratified by sex was constrained by the limited number of osteoporosis events in each stratum (n = 21 men and n = 15 women), restricting the statistical power available for such estimates. Sixth, serum testosterone and dedicated assessment of male hypogonadism (andropause) were not captured in this retrospective dataset; given the small but non-trivial proportion of male patients with otherwise unexplained osteoporosis in our cohort, this represents a recognized residual confounder that should be specifically addressed in prospective external-validation cohorts. Seventh, the duration of chronic hepatitis B (i.e., time since first HBV diagnosis) was not consistently available for the full cohort and was therefore not included as a candidate predictor; the duration of TDF exposure was retained as the most reliable available proxy for cumulative antiviral burden but does not fully capture the biological effect of disease duration before treatment initiation. Eighth, the absolute number of osteoporosis events in this single-center cohort (n = 36) is comparatively small, which constrains both the precision of estimated effect sizes for individual predictors and the stability of subgroup-level inferences derived from ROC and decision-curve outputs (Figure 4 and Figure 6). The optimism-corrected internal validation results should therefore be interpreted as a starting point for external validation rather than a definitive estimate of generalizable model performance, and multicenter cohorts with substantially larger osteoporosis event counts will be required to refine the model’s discriminatory and decision-analytic performance estimates.

## 5. Conclusions

In conclusion, we present a nomogram that offers a clinically viable, methodologically robust tool for predicting TDF-associated osteoporosis in patients with chronic hepatitis B. By incorporating disease-specific inflammatory and comorbidity parameters identified via penalized regression, the model demonstrates superior net clinical benefit over FRAX in guiding DXA screening decisions. Prospective, multicenter validation is required before widespread implementation; however, integration of this tool into electronic clinical workflows could facilitate more precise patient management, optimize healthcare resource allocation, and ultimately reduce the burden of fragility fractures in this vulnerable population.

## Figures and Tables

**Table 1:** Baseline Characteristics of the Study Population Stratified by Osteoporosis Status.

Variable	No Osteoporosis (N = 201)	Osteoporosis (N = 36)	*p*-Value
**Demographics**			
Age (years)	48.3 ± 10.4	64.6 ± 9.7	<0.001
Male sex, n (%)	94 (46.8)	21 (58.3)	0.272
BMI (kg/m^2^)	26.3 ± 2.2	23.3 ± 2.1	<0.001
Smoking, n (%)	94 (46.8)	18 (50.0)	0.860
CCI score	0.7 ± 1.0	2.5 ± 1.2	<0.001
Menopausal status, n (%)	44 (21.9)	14 (38.9)	0.048
Fracture history, n (%)	14 (7.0)	18 (50.0)	<0.001
**HBV-Specific Variables**			
HCV co-infection, n (%)	13 (6.5)	9 (25.0)	0.001
TDF duration (months)	40.8 ± 26.5	85.0 ± 27.9	<0.001
HBV DNA suppressed, n (%)	196 (97.5)	36 (100.0)	0.744
**Laboratory Parameters**			
AST (U/L)	36.9 ± 12.8	47.5 ± 11.7	<0.001
ALT (U/L)	42.1 ± 15.7	53.1 ± 13.0	<0.001
ALP (U/L)	106.6 ± 40.8	179.7 ± 49.3	<0.001
Vitamin D (ng/mL)	25.9 ± 9.1	14.5 ± 6.8	<0.001
eGFR (mL/min/1.73 m^2^)	94.6 ± 18.3	67.2 ± 16.9	<0.001
SII	482.8 ± 75.9	600.7 ± 118.7	<0.001
**Medications**			
Corticosteroid use, n (%)	14 (7.0)	13 (36.1)	<0.001
PPI use, n (%)	54 (26.9)	30 (83.3)	<0.001

Data presented as mean ± standard deviation or n (%). BMI, body mass index; CCI, Charlson Comorbidity Index; HCV, hepatitis C virus; TDF, tenofovir disoproxil fumarate; HBV, hepatitis B virus; AST, aspartate aminotransferase; ALT, alanine aminotransferase; ALP, alkaline phosphatase; eGFR, estimated glomerular filtration rate; SII, Systemic Immune-Inflammation Index; PPI, proton pump inhibitor.

**Table 2:** LASSO-Selected Predictors: Unpenalized Multivariable Logistic Regression Coefficients and Odds Ratios.

Predictor	Coefficient (Beta)	Odds Ratio	95% CI	*p*-Value
Fracture history	2.438	11.45	4.82–27.15	<0.001
CCI score	0.372	1.45	1.15–1.85	0.002
ALP (U/L)	0.030	1.03	0.98–1.08	0.214
Intercept	−5.859			

Variables were first selected by LASSO regression with 10-fold cross-validation (1-SE rule) and subsequently fitted into an unpenalized logistic regression model to obtain interpretable odds ratios. ALP was retained in the nomogram based on its contribution to global model discrimination rather than its standalone *p*-value. CI, confidence interval; CCI, Charlson Comorbidity Index; ALP, alkaline phosphatase.

**Table 3:** Nomogram Performance Metrics: Apparent and Bootstrap-Corrected Internal Validation (N = 237).

Metric	Value
**Discrimination**	
Apparent C-index	0.860
Optimism-corrected C-index (bootstrap, B = 200)	0.845
**Calibration**	
Calibration slope (ideal = 1.00)	0.94
Calibration intercept (ideal = 0.00)	0.02
Brier score	0.095
Nagelkerke R^2^	0.52
**Classification performance at threshold 0.15**	
Sensitivity	0.860
Specificity	0.780
Positive predictive value	0.420
Negative predictive value	0.970
Accuracy	0.790
F1 score	0.560

C-index, concordance index (mathematically equivalent to area under the receiver operating characteristic curve for binary outcomes); B, number of bootstrap replications. Optimism-corrected metrics were derived using bootstrap resampling with 200 replications applied to the full cohort.

**Table 4:** Confusion Matrix at the 0.15 Probability Threshold (Full Cohort, N = 237).

	Predicted: No Osteoporosis	Predicted: Osteoporosis
Actual: No Osteoporosis	157 (True Negative)	44 (False Positive)
Actual: Osteoporosis	5 (False Negative)	31 (True Positive)

## Data Availability

The datasets generated and analyzed during the current study are not publicly available due to institutional data protection regulations but are available from the corresponding author upon reasonable request.

## Supplementary Materials

The following supporting information can be downloaded at: https://www.mdpi.com/article/10.3390/jcm15124442/s1, Table S1: Variance Inflation Factors for All Candidate Variables Considered for LASSO Regression; Table S2: TRIPOD Checklist: Prediction Model Development and Validation.

## Abbreviations

The following abbreviations are used in this manuscript:

ALP	Alkaline Phosphatase
ALT	Alanine Aminotransferase
APRI	AST-to-Platelet Ratio Index
AST	Aspartate Aminotransferase
AUC	Area Under the Curve
BMD	Bone Mineral Density
BMI	Body Mass Index
CCI	Charlson Comorbidity Index
CHB	Chronic Hepatitis B
CI	Confidence Interval
DCA	Decision Curve Analysis
DXA	Dual-Energy X-ray Absorptiometry
eGFR	Estimated Glomerular Filtration Rate
EPV	Events Per Variable
FIB-4	Fibrosis-4 Index
FRAX	Fracture Risk Assessment Tool
HBeAg	Hepatitis B e-Antigen
HBV	Hepatitis B Virus
HCV	Hepatitis C Virus
HIV	Human Immunodeficiency Virus
IRB	Institutional Review Board
LASSO	Least Absolute Shrinkage and Selection Operator
OR	Odds Ratio
PPI	Proton Pump Inhibitor
PTH	Parathyroid Hormone
ROC	Receiver Operating Characteristic
SII	Systemic Immune-Inflammation Index
TAF	Tenofovir Alafenamide
TDF	Tenofovir Disoproxil Fumarate
TRIPOD	Transparent Reporting of a multivariable prediction model for Individual Prognosis or Diagnosis
VIF	Variance Inflation Factor
WHO	World Health Organization
