# Supplementary material for: A Nomogram for Predicting Tenofovir-Associated Osteoporosis in Chronic Hepatitis B
Source: J Clin Med. 2026 Jun 9;15(12):4442. doi: 10.3390/jcm15124442 (PMC13301623; doi:10.3390/jcm15124442)
Supplement: Supplementary file 1 [file jcm-15-04442-s001.zip › Table S1.pdf]

Supplementary Table S1. Variance Inflation Factors for All Candidate Variables Considered for LASSO Regression

**Variable**	**VIF**
Age	5.42
TDF exposure duration	4.15
eGFR	3.88
Charlson Comorbidity Index	3.45
PPI use	2.84
25-Hydroxyvitamin D	2.65
Alkaline phosphatase	2.12
Body mass index	1.85
Systemic Immune-Inflammation Index	1.68
HCV co-infection	1.45
Menopausal status	1.32
Sex (male)	1.32
Fracture history	1.15
**Mean VIF**	**2.56**

VIF values were derived from a full multivariable logistic regression model fitted on all 13 candidate variables before LASSO-based variable selection. The conventional threshold for concerning collinearity is VIF > 5, with VIF > 10 indicating severe multicollinearity. All values in this analysis fell below the threshold of severe collinearity, confirming the appropriateness of the candidate set for penalized regression. eGFR, estimated glomerular filtration rate; TDF, tenofovir disoproxil fumarate; PPI, proton pump inhibitor; HCV, hepatitis C virus; VIF, variance inflation factor.
